# Unusual Indications of Teeth Transplantation: A Literature Review

**DOI:** 10.7759/cureus.29030

**Published:** 2022-09-11

**Authors:** Nuraldeen M Al-Khanati, Ahmad Albassal, Zafin Kara Beit

**Affiliations:** 1 Department of Oral and Maxillofacial Surgery, Faculty of Dental Medicine, Damascus University, Damascus, SYR

**Keywords:** oral rehabilitation, impacted teeth, dental transplantation, teeth replacement therapy, oral surgery

## Abstract

Dental implants are one of the best valid tooth replacement options, though these are not always appropriate in growing young patients. Tooth autotransplantation can be indicated then. However, this is not the only scenario where dental transplantation can be indicated. This comprehensive literature review discusses a wide range of unusual indications of dental transplantation as reported throughout the medical literature. Surprisingly, these indications include management of some developmental dental anomalies, hypodontia, oroantral communications, alveolar clefts, deficient alveolar ridges, ectopic teeth, and maxillofacial injuries. Limited high-quality evidence in this field regarding most of these unusual indications warrants further research of high-quality design.

## Introduction and background

Tooth transplantation is a surgical biological way of managing tooth loss and may have multiple advantages over traditional other options of replacing missing teeth, especially in children and adolescents. Despite the predictable high success rates proven in the literature, transplantation of teeth does not receive the acceptance it deserves among clinicians [1]. The survival and success rates of tooth transplantation can exceed 90% after mid-term and long-term follow-up periods [2-4]. Many factors can influence the outcomes of dental transplantation [5]. This surgical procedure must be done with full understanding of success-related biological concepts and their impact on clinical transplantation techniques. Gentle surgical technique that preserves intact transplant’s periodontal ligament is the key to successful transplantation [6].

Most of the published literature regarding transplantation of teeth roughly confines its indications to one of the two most common clinical scenarios. The first is heavily destructed non-restorable permanent molar in a young patient with the presence of suitable non-functional impacted or partially erupted third molar donor. In this situation, autotransplantation of the third molar into the first or second molar site can be a very predictable treatment. The second most common clinical indication of tooth transplantation is the management of traumatically avulsed teeth of the anterior maxilla [7]. In this case, a multidisciplinary approach is inevitable. Orthodontists, pediatric dentists, and oral surgeons can endorse autotransplantation of one of the premolars as a donor to replace the lost maxillary incisor. Mandibular premolars are usually considered appropriate donors. Either fixed crown or direct composite restoration is needed later on to achieve esthetics, and posterior space closure is usually performed via sequential stages of an orthodontic treatment plan. Nevertheless, transplantation of teeth has much wider indications than the aforementioned. This article presents a literature review and highlights the unusual clinical situations where tooth transplantation can be indicated.

## Review

Alternative to dental implants

One of the major limitations of the use of dental implants from patients’ perspective, especially in low- and middle-income countries, is its high costs [8,9]. Furthermore, dental implants procedure is a matter of controversy in childhood and adolescence. Autotransplantation of teeth, when applicable, can provide an affordable alternative to dental implants [1]. Prognoses of transplanted teeth and osseointegrated dental implants are comparable [10]. Yet, successful autotransplanted teeth can have more advantages in comparison to osseointegrated implants that lack periodontal ligament. This allows physiological micromovement and works as a damper reducing functional stresses applied to a successfully transplanted tooth. Also, functioning vital periodontium can provide proprioception and thermal feedback, and allow orthodontic treatment [1]. Moreover, dental implants and peri-implant tissues are more susceptible to bacterial and disease progression [11,12].

Lesions at the recipient site

Many publications stated that the recipient site must be free from any pathological activity in order to achieve successful transplantation [13,14]. At any rate, pioneering works exist that indicate the possibility of immediate dental transplantation into lesion-associated recipient sites after being managed appropriately [12,15,16]. A successful immediate tooth transplantation case with signs of chronic odontogenic infection at the recipient site (e.g., intraoral sinus tract) has been reported recently [12]. The key is to debride and heavily irrigate the recipient bed prior to autotransplantation. Manuka honey application as an anti-microbial agent was also reported during management of transplant bed in such cases [15]. Keranmu et al. performed a clinical study including 52 participants and concluded that concentrated growth factors application into the transplant bed with chronic periapical lesions could improve healing and increase transplantation success rates [16]. Alkofahi et al. supposed that platelet-rich fibrin could have a role in successful healing and continuous root development of the immature transplants [17].

Space maintainer

Space maintenance after teeth loss is necessary until the achievement of complete dentofacial growth. Dental autotransplantation was proposed to be used as a space-maintaining method in growing subjects [18]. Although It can be provided as a definitive treatment with predicted long-term survival in most cases, partial or complete failures of autologous dental transplants (e.g., due to ankylosis, root resorption, nonfunctional transplant in infraocclussal position, etc.) when occurred do not prevent or interfere with the subsequent prosthetic treatment. Contrariwise, the transplanted tooth can provide space maintenance and alveolar process preservation, even if it is eventually lost [19]. Plakwicz et al. indicated that transplantation of premolars replacing central incisor allowed alveolar bone preservation in the maxilla over long‐term follow-up periods [20]. Sönmez et al. reported an unusual case in which they considered autotransplantation of a primary canine as a temporary space maintainer in an eight-year-old child who lost her permanent maxillary central incisor [21].

Hypodontia

Another indication for dental autotransplantation is congenital tooth absence [7]. Congenitally missing teeth leave one or more edentulous spaces that are usually closed by orthodontic treatment or left for later prosthetic management [22]. Szemraj-Folmer et al. reported an interesting case of asymmetric multiple hypodontia in an eight-year-old girl treated successfully with autotransplantation of two developing premolars [23]. Mandibular right second premolar and maxillary right first premolar were transplanted to surgically created sockets between the first molar and central incisor in the left maxilla [23]. In combination with orthodontic alignment and adhesive fiber-reinforced bridge, this treatment established good function without the need for dental implants placement during around six years of follow-up after dental transplantation [23].

Regional odontodysplasia

Regional odontodysplasia is a rare developmental disorder characterized by hypomineralization and hypoplasia of dentin and enamel, mostly involving both primary and permanent dentitions [24]. It usually affects teeth of one quadrant, which appear as shadowy yellow “ghost teeth” with recurrent purulent gingivitis [24,25]. Extraction of affected teeth in early age will lead to extensive resorption of the alveolar bone. Thus, treatment must include preservation of the alveolar process. This can be achieved by transplantation of teeth from the unaffected quadrants. Fortunately, regional odontodysplasia only affects dental hard tissues rather than the bone; therefore, autogenous transplantation of teeth can offer a viable treatment option when donor teeth are available [26]. In Denmark, a 13-year-old patient with regional odontodysplasia was managed by transplanting teeth 15 and 25 to the affected region, followed by orthodontic treatment [27]. However, one of the transplanted teeth was extracted after three years due to root resorption [27]. Another case was also treated with autotransplantation of premolars at age 10 with satisfactory outcome over a five-year follow-up period [26]. Ziegler and Neukam transplanted the right and left maxillary second premolars in a two-step procedure into the affected quadrant, and obtained good functional occlusion via this treatment modality [25].

Unerupted teeth

Dental autotransplantation can be indicated for the purpose of surgical repositioning of an impacted or ectopic permanent teeth, especially canines. In most cases, impacted and ectopically erupted canines are surgically exposed and repositioned via orthodontic approach [28,29]. In certain situations, surgical exposure and subsequent orthodontic traction to realign the impacted tooth are very difficult or nearly impossible due to severe ectopic impaction position. Sometimes, patients refuse orthodontic approach as it entails significantly increased treatment time and costs caused by the presence of canine impaction [30]. In such scenarios, canine transplantation can offer a worthy option to restore its natural orientation in a faster, more expedient way [14,31]. Zufía et al. reported an autotransplantation case of impacted maxillary canine combined with guided tissue regeneration, guided bone regeneration, and orthodontic treatment [32]. After four years, the transplanted canine was functioning with good esthetic results [32]. According to Grisar et al., autogenous transplantation of impacted maxillary canines may show successful results for up to 21 years post-transplantation [33]. Unerupted supernumerary teeth can also be considered as potential donors for dental autotransplantation [34]. Pang et al. presented an interesting case report of a 13-year-old male with a fully impacted maxillary second premolar that was transplanted from its ectopic position into its original site [35]. The transplantation procedure was preceded by maxillary sinus floor lifting and allogenic bone augmentation with favorable outcomes for two years of follow-up [35].

Oroantral communication

Oroantral communication is a possible complication of dental extraction in the posterior maxilla [36]. Many techniques have been introduced in the management of this complication. These usually include soft tissue grafts and flaps, such as palatal rotational and buccal advancement flaps. An unusual indication of transplantation of teeth is the use of transplanted tooth in the management of oroantral communication. Many case reports showed successful closure of oroantral communication with immediate implementation of autogenous third molar transplantation into the site of oroantral opening [37,38]. Transplantation of third molars with completed root formation and closed apices was also reported to be successful and promising in the management of oroantral communications [39]. Moreover, evidence of maxillary sinus floor lifting after closure of the communication was reported and could be a great advantage of this technique due to the regenerative potential of transplant’s vital periodontal ligament [40]. Assad et al. presented results of one-year follow-up clinical study showing a success rate of 95% in closing oroantral communications with third molar transplantations [41]. They noted that this technique was not preferable in extensive oroantral opening [41].

Bone regeneration

This is an important indication where deficient alveolar dimension exists. A complete alveolar bone regeneration following dental autotransplantation was recently highlighted [6,42]. Autotransplantation of a developing lower premolar to replace severely traumatized upper central incisor resulted in satisfactory outcomes with complete regeneration of normal alveolus [42]. This is directly related to bone-inductive potential of the periodontal ligament [6]. Another similar case was presented by Waldon et al. [43]. Three years after tooth injury episode, infra-occluded root-resorbed permanent central incisor was treated with autotransplantation of immature mandibular premolar [43]. Continued premolar root development was observed, and this was associated with alveolar process regeneration and complete eruption of the transplanted tooth [43]. Preservation of the alveolar bone after successful autotransplantation was reported over long-term follow-ups [20]. In this context, a derivative of enamel matrix, i.e., Emdogain®, claimed to stimulate differentiation of osteoblasts and proliferation of pre-odontoblast, was tested by topical application into the transplanted tooth root [44]. However, Marques-Ferreira et al. found no differences as compared to the control group, i.e., saline solution [44].

Clefts

Cleft lip, alveolus, and palate are often associated with dental anomalies and malocclusions [45-47]. Many dental disturbances can be created by the cleft area including impacted teeth, supernumerary teeth, microdontia, and hypodontia [46,47]. The maxillary lateral incisor is the most affected tooth by the cleft defect [46]. Treatment protocols should not only include osteoplasty, which can successfully close the alveolar cleft defect, but also include management of missing teeth. Here, an unusual indication of tooth autotransplantation arises. Aizenbud et al. described their experience transplanting incompletely developed mandibular premolar teeth into grafted alveolar clefts [48]. They showed long-term satisfactory results of this procedure as a part of cleft patient’s treatment plan [48]. Jayasuriya et al. presented a case of a 10-year-old cleft patient treated with bone grafting from tibia, platelet-rich plasma, and lateral incisor autotransplantation simultaneously [49]. The case was followed up for three years and showed acceptable clinical outcomes [49]. Schaaf et al. recommended this treatment modality in young patients with cleft lip and alveolus early in time before bone augmentation [50]. Autotransplantation of teeth was also described in a patient who had Pierre-Robin syndrome including cleft lip and palate [43]. An upper permanent canine was considered as donor and transplanted to replace a dilacerated central incisor. The transplanted canine was followed up for two years and showed normal function and healthy periodontal tissues [43].

Medically compromised patients

Many publications in the literature concerning transplantation of teeth assume that patient selection must ensure good general health with no medical issues, otherwise dental autotransplantation will be contraindicated [14,51,52]. Autotransplantation of teeth should be avoided in uncontrolled systemic conditions and endocrine disorders that may contribute to possible root resorption, e.g., hyperparathyroidism, calcinosis, Gaucher's disease, and Paget's disease [53]. Medical evaluation of every patient is, for sure, an important step in the treatment planning. Yet, there is lack of evidence and studies that support the claim that autotransplantation of teeth in all medically compromised patients cannot be indicated. In dental treatments, risks and benefits should always be evaluated, and, in many situations, benefits overweigh the risk of complications in medically compromised patients [54]. Dental implants are much more commonly performed in dental clinics than dental transplantation. Thus, studies have detailed outcomes of dental implants in different medical conditions [55], while there is insufficient evidence in the case of dental autotransplantation. Furthermore, most studies regarding transplantation of teeth included only healthy patients; medically compromised patients and even smokers are usually excluded from studies’ samples in order to achieve sample homogeneity [7,56,57]. Nevertheless, few papers reported dental autotransplantation in individuals who were diagnosed with diabetes mellitus and who had smoking habits [58-60]. Smoking was found to have an insignificant effect on 5-year and 10-year survival of dental autotransplantation according to Yoshino et al. [60]. Successful autotransplantation of teeth was reported in patients with controlled type I and type II diabetes [58,59]. Thus, further investigations and more studies are warranted.

En bloc autotransplantation

Krasny et al. have introduced a novel technique of dental transplantation, namely “en bloc autotransplantation” [61-63]. This surgical technique involves dissecting a bone block that includes the donor tooth within it and transplanting them as one block into the recipient site. This method was proposed to preserve the periodontal ligament and bone allowing revascularization and growth continuation of the transplanted tooth [61]. Surgical bone incisions are preferably made by means of surgical piezoelectric methods to minimize potential complications as this technique is more complicated than normal transalveolar transplantation [61]. Guided bone regeneration via bone allografts and the use of platelet-rich fibrin can be implemented simultaneously with en bloc transplantation in the recipient site [62]. Based on Kransy et al.’s results, risk of transplanted tooth root ankylosis is negligible; however, it does not completely prevent the risk of external root resorption [61]. This method is mainly indicated when less complicated techniques fail, e.g., in cases of severe alveolar bony defect at the recipient site.

Jaw reconstruction

Various jaw pathologies (e.g., chronic infections, bone necrosis, and tumors) can often be treated with segmental resection [64]. Jaw resection results in severe functional impairments that require rehabilitation. Treatment goals include reconstruction of the jaw discontinuity defect (usually through autogenous bone grafts from iliac crest, fibula, radius, etc.) and rehabilitation of masticatory function [64,65]. As such, another indication of teeth autotransplantation can be presented. In 2008, Landes et al. reported unconventional mandibular reconstruction of jaw defect resulting from resecting recurrent myxoma in the jaw of a 14-year-old male [66]. Free autogenous iliac crest block was used for bone reconstruction, followed by autotransplantation of three third molars into the grafted area five months later [66]. Relative success was reported withtwo2 surviving transplanted teeth after a follow-up period of 30 months [66]. A similar case of recurrent odontogenic myxoma in the maxilla was reported by Friedrich et al. [67]. The 15-year-old patient was managed with partial maxillectomy, bone reconstruction with iliac crest graft, and transplantation of the patient’s upper third molars to the affected second premolar and first molar region two months later [67]. The transplanted teeth survived for three years until recurrence was unfortunately detected and included in a complete lateral maxilla resection [67]. We suggest that immediate en bloc autotransplantation of teeth could be indicated and would provide an acceptable treatment choice in cases of less aggressive lesions that exhibit less recurrent behavior.

Transplantation of cryopreserved teeth

Indications of autotransplantation of teeth are usually limited to cases where a suitable donor tooth is available at the time of replacing missing or destructed tooth. Dental extractions of intact impacted, supernumerary, and even erupted teeth (e.g., premolars extraction for orthodontic reasons) are often performed when there is no need for tooth replacement therapy in both jaws. This need may appear later in life, but dental autotransplantation option cannot be performed then due to a lack of suitable donor. Hence, it is important to find a possible way to preserve the tooth, its periodontal ligament, and pulp tissues for a later time to be used when it is needed. In vitro, Temmerman et al. found that standard cryopreservation procedure under controlled conditions does not affect the human periodontal ligament cells viability nor their capacity to proliferate and differentiate [68]. Another in vitro study indicated that storage temperature has no effect on cryopreserved teeth fracture behavior from a physical perspective [69]. More interestingly, results of Lee et al.’s work indicated that cryopreserved teeth can maintain pulp stem cell growth potential, surface markers, and differentiation ability [70]. These results suggested that dental autotransplantation can be performed after whole teeth cryopreservation [70]. A systematic review in 2010 also highlighted the potential clinical application of cryopreserved donor teeth for autogenous transplantation [71]. It was suggested that endodontic therapy of these teeth could provide better success [71]. Later on, Yoshizawa et al. presented clinical and radiographic outcomes of seven cases of autogenous transplantation of cryopreserved teeth that had been preserved from 4 to 36 months pre-transplantation [72]. Risk of replacement external root resorption of the transplanted teeth exists after cryopreservation, as four (57%) out of the seven cases exhibited this complication [72]. However, the remaining three cases were considered successful, indicating the potential clinical use of this method [72]. Kaku et al. had developed Cells Alive System (CAS), a programmed freezer with a novel magnetic field for the purpose of cryopreservation of teeth [73]. In 2015, they reported a three-year follow-up successful transplanted premolar that had been cryopreserved with CAS for six years [73]. The transplant showed healthy periodontal tissues, lamina dura, and bone regeneration with no inflammatory or replacement root resorption three years after transplantation [73]. These clinical reports, although very few, may illuminate the promising potential indication of autogenous dental transplantation with the implementation of novel technologies of cryopreservation.

Allotransplantation of teeth

The bulk of published literature on dental transplantation describes autogenous transplantation of teeth within various conditions and indications as a reliable treatment option [1-7,12-21]. Allogenic dental transplantation cases, where donor and recipient are two different individuals, are scarce in the medical literature due to several reasons. Risk of immunological rejection, cross-infections, ankylosis, root resorption, and/or tooth loss seems high and indicates poor prognosis of allotransplantation of teeth [74-76]. Furthermore, opportunistic infections, systemic toxicity, and metabolic complications associated with immunosuppression therapy pose additional risks [77]. Schwartz et al. attempted to modulate the immune reaction toward the allogenic transplanted teeth in monkeys through preoperative donor-specific blood transfusions but found no significant effect of this method in reducing ankylosis of allotransplanted teeth [78]. The inflammatory root resorption seems to be the most prominent manifestation of immunological rejection of allogenic dental transplant [79]. Cross-contamination is another ethical issue that can collide with the implementation of this method. Abe et al. reported a case of a 45-year-old male who had infectious endocarditis two weeks after allogenic dental transplantation [80]. Schuman et al. reported cadaveric allotransplantation of molar tooth into the jaw of a 21-year-old male with three-year survival that ended eventually with ankylosis and root resorption [76]. Even when dental allotransplantation was performed between close family members (i.e., mother and daughter), replacement root resorption resulted [81]. In contrast, allotransplantation of teeth was reported to be safe and successful in a few reports [82,83]. Donor blood testing for HBV, HCV, and HIV was mandatory, and transplant was preserved in disinfectants, i.e., chlorhexidine solution after extraction to avoid cross-contamination [83]. We believe that allogenic dental transplantation is indicated and more justified in more complicated cases than in single tooth transplantation scenarios. Facial transplantation can be defined as allogenic composite tissue transplantation involving teeth, bone, muscles, mucosa, and skin of the face [84]. Immunosuppressive therapy is necessary to eliminate risk of tissue rejection after transplantation. In 2015, a team from Turkey reported successful facial allotransplantation including seven teeth, the maxilla, nose, and upper lip from a heart-beating brain-dead human cadaver with a follow-up of two and half years [84]. In 2019, Ramly et al. reported two patients with severe facial injuries treated successfully with allogenic facial, maxillomandibular, and dental transplantations [77]. Such cases confirm the validity of allogenic transplantation as a reconstructive treatment modality in situations where autogenous reconstruction is not available.

## Conclusions

Dental transplantation seems to have a wider range of indications than what is common between oral surgeons and dental practitioners. These indications include replacement of permanent teeth associated with poor prognosis, replacement of developmentally missing teeth, management of alveolar clefts, repositioning impacted or ectopic teeth, management of oroantral communications, autotransplantation of deciduous teeth as space maintainers, and cases of maxillomandibular reconstructions. More attention should be given to this treatment modality among clinicians and researchers. There is an imperative need for conducting well-designed studies to reach reliable conclusions regarding the unusual indications of dental transplantations.

## References

[1] Al-Khanati NM, Beit ZK (2021). Is dental autotransplantation underestimated and underused by Syrian dentists?. J Educ Eval Health Prof.

[2] Raabe C, Bornstein MM, Ducommun J, Sendi P, von Arx T, Janner SF (2021). A retrospective analysis of autotransplanted teeth including an evaluation of a novel surgical technique. Clin Oral Investig.

[3] van Westerveld KJ, Verweij JP, Toxopeus EE, Fiocco M, Mensink G, van Merkesteyn JP (2019). Long-term outcomes 1-20 years after autotransplantation of teeth: clinical and radiographic evaluation of 66 premolars and 8 molars. Br J Oral Maxillofac Surg.

[4] Kokai S, Kanno Z, Koike S, Uesugi S, Takahashi Y, Ono T, Soma K (2015). Retrospective study of 100 autotransplanted teeth with complete root formation and subsequent orthodontic treatment. Am J Orthod Dentofacial Orthop.

[5] Al-Khanati NM, Kara Beit Z (2022). Should we predict poor prognosis in autotransplantation of teeth with completed root formation?. Ann Med Surg (Lond).

[6] Park JH (2022). Autotransplantation and alveolar bone regeneration. AJO-DO Clinical Companion.

[7] Nimčenko T, Omerca G, Varinauskas V, Bramanti E, Signorino F, Cicciù M (2013). Tooth auto-transplantation as an alternative treatment option: A literature review. Dent Res J (Isfahan).

[8] Siddique EA, Bhat PR, Kulkarni SS, Trasad VA, Thakur SL (2019). Public awareness, knowledge, attitude and acceptance of dental implants as a treatment modality among patients visiting SDM College of Dental Sciences and Hospital, Dharwad. J Indian Soc Periodontol.

[9] Arora K Jr, Kaur N 2nd, Kaur G 3rd, Garg U 4th (2022). Knowledge, awareness, and attitude in using dental implants as an option in replacing missing teeth among dental patients: survey-based research in a dental teaching hospital in Derabassi, Punjab. Cureus.

[10] Rohof EC, Kerdijk W, Jansma J, Livas C, Ren Y (2018). Autotransplantation of teeth with incomplete root formation: a systematic review and meta-analysis. Clin Oral Investig.

[11] Ivanovski S, Lee R (2018). Comparison of peri-implant and periodontal marginal soft tissues in health and disease. Periodontol 2000.

[12] Al-Khanati NM, Kara Beit Z (2022). Reconsidering some standards in immediate autotransplantation of teeth: case report with 2-year follow-up. Ann Med Surg (Lond).

[13] Martin K, Nathwani S, Bunyan R (2018). Autotransplantation of teeth: an evidence-based approach. Br Dent J.

[14] Algubeal HM, Alanazi AF, Arafat AS, Fatani B, Al-Omar A (2022). Autotransplantation of the lower posterior teeth: a comprehensive review. Cureus.

[15] Al-Khanati NM (2019). Immediate autotransplantation of immature maxillary third molar: a case report with 4-year clinical and radiographic follow-up. Saudi Endod J.

[16] Keranmu D, Ainiwaer A, Nuermuhanmode N, Ling W (2021). Application of concentrated growth factor to autotransplantation with inflammation in recipient area. BMC Oral Health.

[17] Alkofahi H, Maghaireh A, Fnaish M, Jarrah M, Bataineh M (2020). Application of platelet-rich fibrin as regeneration assistant in immediate auototransplantation of third molar with unformed roots: case report and review of literature. Case Rep Dent.

[18] Saccomanno S, Laganà D, Saran S, De Stefani A, Pirelli P, Bruno G, Gracco A (2021). Proposal of use of the autotransplantation of the third molar as space maintainer in growing patients: a review of literature and a clinical case. J Biol Regul Homeost Agents.

[19] Verweij JP, Wes JT, van Teeseling RA, Becking AG (2021). Pre-autotransplantation alveolar process augmentation and premolar autotransplantation as a treatment method for single tooth replacement in adolescents. Int J Oral Maxillofac Surg.

[20] Plakwicz P, Andreasen JO, Górska R, Burzykowski T, Czochrowska E (2021). Status of the alveolar bone after autotransplantation of developing premolars to the anterior maxilla assessed by CBCT measurements. Dent Traumatol.

[21] Sönmez D, Dalci K, Tunç ES (2008). Treatment of an avulsed maxillary permanent central incisor by autotransplantation of a primary canine tooth. Int Endod J.

[22] Rakhshan V (2015). Congenitally missing teeth (hypodontia): a review of the literature concerning the etiology, prevalence, risk factors, patterns and treatment. Dent Res J (Isfahan).

[23] Szemraj-Folmer A, Kuc-Michalska M, Plakwicz P (2019). Patient with asymmetric multiple hypodontia treated with autotransplantation of 2 premolars. Am J Orthod Dentofacial Orthop.

[24] Nijakowski K, Woś P, Surdacka A (2022). Regional odontodysplasia: a systematic review of case reports. Int J Environ Res Public Health.

[25] Ziegler S, Neukam FW (2012). Regional odontodysplasia: orthodontic treatment and transplantation of premolars. Am J Orthod Dentofacial Orthop.

[26] Cahuana A, González Y, Palma C (2005). Clinical management of regional odontodysplasia. Pediatr Dent.

[27] Hess P, Lauridsen EF, Daugaard-Jensen J, Worsaae N, Kofod T, Hermann NV (2020). Treatment strategies for patients with regional odontodysplasia: a presentation of seven new cases and a review of the literature. Oral Health Prev Dent.

[28] Manne R, Gandikota C, Juvvadi SR, Rama HR, Anche S (2012). Impacted canines: etiology, diagnosis, and orthodontic management. J Pharm Bioallied Sci.

[29] Thirunavukkarasu R, Sriram G, Satish R (2015). The orthodontic management of ectopic canine. J Pharm Bioallied Sci.

[30] Barlow ST, Moore MB, Sherriff M, Ireland AJ, Sandy JR (2009). Palatally impacted canines and the modified index of orthodontic treatment need. Eur J Orthod.

[31] Park JH, Tai K, Hayashi D (2010). Tooth autotransplantation as a treatment option: a review. J Clin Pediatr Dent.

[32] Zufía J, Abella F, Gómez-Meda R, Blanco H, Roig M (2020). Autotransplantation of impacted maxillary canines into surgically modified sockets and orthodontic treatment: a 4-year follow-up case report. Int J Esthet Dent.

[33] Grisar K, Nys M, The V, Vrielinck L, Schepers S, Jacobs R, Politis C (2019). Long-term outcome of autogenously transplanted maxillary canines. Clin Exp Dent Res.

[34] Tirali RE, Sar C, Ates U, Kizilkaya M, Cehreli SB (2013). Autotransplantation of a supernumerary tooth to replace a misaligned incisor with abnormal dimensions and morphology: 2-year follow-up. Case Rep Dent.

[35] Pang NS, Choi YK, Kim KD, Park W (2011). Autotransplantation of an ectopic impacted premolar with sinus lift and allogenic bone graft. Int Endod J.

[36] Shahrour R, Shah P, Withana T, Jung J, Syed AZ (2021). Oroantral communication, its causes, complications, treatments and radiographic features: A pictorial review. Imaging Sci Dent.

[37] Lucidarme Q, Deshors A, Lescaille G, Baaroun V, Mondoloni M (2022). Tooth auto-transplantation to close an oro-sinusal communication using a 3D printed model to adapt the alveolar socket: a case report. J Oral Med Oral Surg.

[38] Nagori SA, Jose A, Bhutia O, Roychoudhury A (2015). A case of oro-antral communication closed by autotransplantation of third molar. J Maxillofac Oral Surg.

[39] Kitagawa Y, Sano K, Nakamura M, Ogasawara T (2003). Use of third molar transplantation for closure of the oroantral communication after tooth extraction: a report of 2 cases. Oral Surg Oral Med Oral Pathol Oral Radiol Endod.

[40] Melillo M, Boschini L (2021). Autotransplantation of third molar for oro-antral communication closure: evidence of sinus elevation after healing. Minerva Dent Oral Sci.

[41] Assad M, Alkhaled M, Alhajj MN (2018). Evaluation of a new surgical technique for closing oroantral fistula using auto-transplanted upper third molar: a 1-year follow-up study. J Maxillofac Oral Surg.

[42] Czochrowska E, Plakwicz P (2022). Alveolar bone regeneration following autotransplantation of immature premolars to replace traumatized maxillary incisor. AJO-DO Clinical Companion.

[43] Waldon K, Barber SK, Spencer RJ, Duggal MS (2012). Indications for the use of auto-transplantation of teeth in the child and adolescent. Eur Arch Paediatr Dent.

[44] Marques-Ferreira M, Rabaça-Botelho MF, Carvalho L, Oliveiros B, Palmeirão-Carrilho EV (2011). Autogenous tooth transplantation: evaluation of pulp tissue regeneration. Med Oral Patol Oral Cir Bucal.

[45] Paradowska-Stolarz A, Kawala B (2014). Occlusal disorders among patients with total clefts of lip, alveolar bone, and palate. Biomed Res Int.

[46] Bezerra BT, Pinho JN, da Silva LC (2017). Tooth abnormalities in individuals with unilateral alveolar clefts: a comparison between sides using cone-beam computed tomography. J Clin Exp Dent.

[47] Vuletić M, Knežević P, Jokić D, Rebić J, Žabarović D, Macan D (2014). Alveolar bone grafting in cleft patients from bone defect to dental implants. Acta Stomatol Croat.

[48] Aizenbud D, Zaks M, Abu-El-Naaj I, Rachmiel A, Hazan-Molina H (2013). Mandibular premolar autotransplantation in cleft affected patients: the replacement of congenital missing teeth as part of the cleft patient's treatment protocol. J Craniomaxillofac Surg.

[49] Jayasuriya NS, Ratnapreya S, Medawela RM, Cooray MP (2022). Simultaneous auto-tooth transplantation of an impacted lateral incisor to the alveolar bone graft site. Ceylon J Sci.

[50] Schaaf H, Kerkmann H, Pitka F, Bock NC, Attia S (2016). Replantation of a displaced incisor in a boy with a cleft lip and alveolus: a case report. J Med Case Rep.

[51] Ong D, Itskovich Y, Dance G (2016). Autotransplantation: a viable treatment option for adolescent patients with significantly compromised teeth. Aust Dent J.

[52] Armstrong L, O'Reilly C, Ahmed B (2020). Autotransplantation of third molars: a literature review and preliminary protocols. Br Dent J.

[53] Ahangari Z, Nasser M, Mahdian M, Fedorowicz Z, Marchesan MA (2015). Interventions for the management of external root resorption. Cochrane Database Syst Rev.

[54] Ghimire P, Suwal P, Basnet BB (2022). Management of medically compromised prosthodontic patients. Int J Dent.

[55] Mozzati M, Gallesio G, Menicucci G, Manzella C, Tumedei M, Del Fabbro M (2021). Dental implants with a calcium ions-modified surface and platelet concentrates for the rehabilitation of medically compromised patients: a retrospective study with 5-year follow-up. Materials (Basel).

[56] Dhar S, Singh G, Mishra M, Gaur A (2022). A prospective study on autotransplantation of mandibular third molars with complete root formation [Online Ahead of Print]. Craniomaxillofac Trauma Reconstr.

[57] Yoshino K, Ishizuka Y, Sugihara N (2013). Gender difference in tooth autotransplantation with complete root formation: a retrospective survey. J Oral Rehabil.

[58] Alhabshi M, Attas S, Melebari L, Parambil M, Kensara J, Alturki M, Abed H (2021). Tooth autotransplantation in a diabetic patient with 2-year follow-up. Gen Dent.

[59] Muto A, Kubokawa K, Kaise K (2013). [A case of autotransplantation of teeth to areas close to the maxillary sinus]. Jpn J Conserv Dent.

[60] Yoshino K, Ishizuka Y, Sugihara N (2014). Risk factors affecting third molar autotransplantation during 5 and 10 years. Bull Tokyo Dent Coll.

[61] Krasny M, Krasny K, Wojtowicz A (2022). Long term outcomes of en-block autotransplantation of a tooth [Online Ahead of Print]. Cell Tissue Bank.

[62] Krasny M, Krasny K, Kamiński A (2020). Alternative methods of repositioning impacted maxillary canines in the dental arch-en bloc autotransplantation of a tooth. Transplant Proc.

[63] Krasny M, Krasny K, Wojtowicz A (2020). En bloc autotransplantation of retained canine in the mandible: a case presentation. Int J Periodontics Restorative Dent.

[64] Kumar VV, Rometsch E, Thor A, Wolvius E, Hurtado-Chong A (2020). Segmental mandibular reconstruction using tissue engineering strategies: a systematic review of individual patient data. Craniomaxillofac Trauma Reconstr.

[65] Petrovic I, Ahmed ZU, Huryn JM, Nelson J, Allen RJ Jr, Matros E, Rosen EB (2019). Oral rehabilitation for patients with marginal and segmental mandibulectomy: a retrospective review of 111 mandibular resection prostheses. J Prosthet Dent.

[66] Landes CA, Glasl B, Ludwig B, Rieger J, Sader R (2008). Tooth autotransplantation in a free iliac crest graft for prosthetic reconstruction. J Craniofac Surg.

[67] Friedrich RE, Scheuer HA, Höltje W (2016). Recurrent maxillary odontogenic myxoma following partial maxillary resection and consecutive osseous reconstruction including tooth transplantation. Anticancer Res.

[68] Temmerman L, Dermaut LR, De Mil M, Van Maele G, Beele H, De Pauw GA (2008). Influence of cryopreservation on human periodontal ligament cells in vitro. Cell Tissue Bank.

[69] Angermair J, Nolte D, Linsenmann R, Kunzelmann KH (2020). The influence of storage temperature on fracture behavior of cryopreserved teeth-An in vitro study. Clin Exp Dent Res.

[70] Lee SY, Chiang PC, Tsai YH, Tsai SY, Jeng JH, Kawata T, Huang HM (2010). Effects of cryopreservation of intact teeth on the isolated dental pulp stem cells. J Endod.

[71] Osathanon T (2010). Transplantation of cryopreserved teeth: a systematic review. Int J Oral Sci.

[72] Yoshizawa M, Koyama T, Izumi N (2014). Autotransplantation or replantation of cryopreserved teeth: a case series and literature review. Dent Traumatol.

[73] Kaku M, Shimasue H, Ohtani J (2015). A case of tooth autotransplantation after long-term cryopreservation using a programmed freezer with a magnetic field. Angle Orthod.

[74] Kim E, Cho SW, Yang JY, Cai J, Lee SL, Ohshima H, Jung HS (2006). Tooth survival and periodontal tissues healing of allogenic-transplanted teeth in the mice. Oral Dis.

[75] Cohen AS, Shen TC, Pogrel MA (1995). Transplanting teeth successfully: autografts and allografts that work. J Am Dent Assoc.

[76] Schuman NJ, Owens BM, Mincer HH (1997). Dental transplants: discussion and case report. J Clin Pediatr Dent.

[77] Ramly EP, Kantar RS, Diaz-Siso JR, Alfonso AR, Shetye PR, Rodriguez ED (2019). Outcomes after tooth-bearing maxillomandibular facial transplantation: insights and lessons learned. J Oral Maxillofac Surg.

[78] Schwartz O, Thomsen M, Dickmeiss E, Andreasen JO (2022). Effect of donor-specific blood transfusions on allotransplanted teeth in a monkey model: histoquantification of periodontal healing. Dent Traumatol.

[79] Schwartz O, Andreasen JO (2002). Allo- and autotransplantation of mature teeth in monkeys: a sequential time-related histoquantitative study of periodontal and pulpal healing. Dent Traumatol.

[80] Abe K, Nakamura S, Ninomiya T (1996). Infective endocarditis caused by Campylobacter fetus after allogeneic tooth transplantation: a case report. Br J Oral Maxillofac Surg.

[81] Xu HD, Miron RJ, Zhang XX, Zhang YF (2019). Allogenic tooth transplantation using 3D printing: A case report and review of the literature. World J Clin Cases.

[82] Revathy V, Suryakanth M, Poornima P, Subba Reddy VV (2012). Allotransplantation of tooth: a case report. J Clin Pediatr Dent.

[83] Pai SM, Patil PS, Poornima P, Subbareddy VV (2013). Mesiodens used for allotransplantation. Eur J Gen Dent.

[84] Özel AŞ, Güçlü ZA, Gülşen A, Özmen S (2015). The importance of the condition of the donor teeth and jaws during allogeneic face transplantation. J Craniofac Surg.

